# Supplementary figures and images for: Microbial occurrence and symbiont detection in a global sample of lichen metagenomes
Source: PLoS Biol. 2024 Nov 7;22(11):e3002862. doi: 10.1371/journal.pbio.3002862 (PMC11542873; doi:10.1371/journal.pbio.3002862)

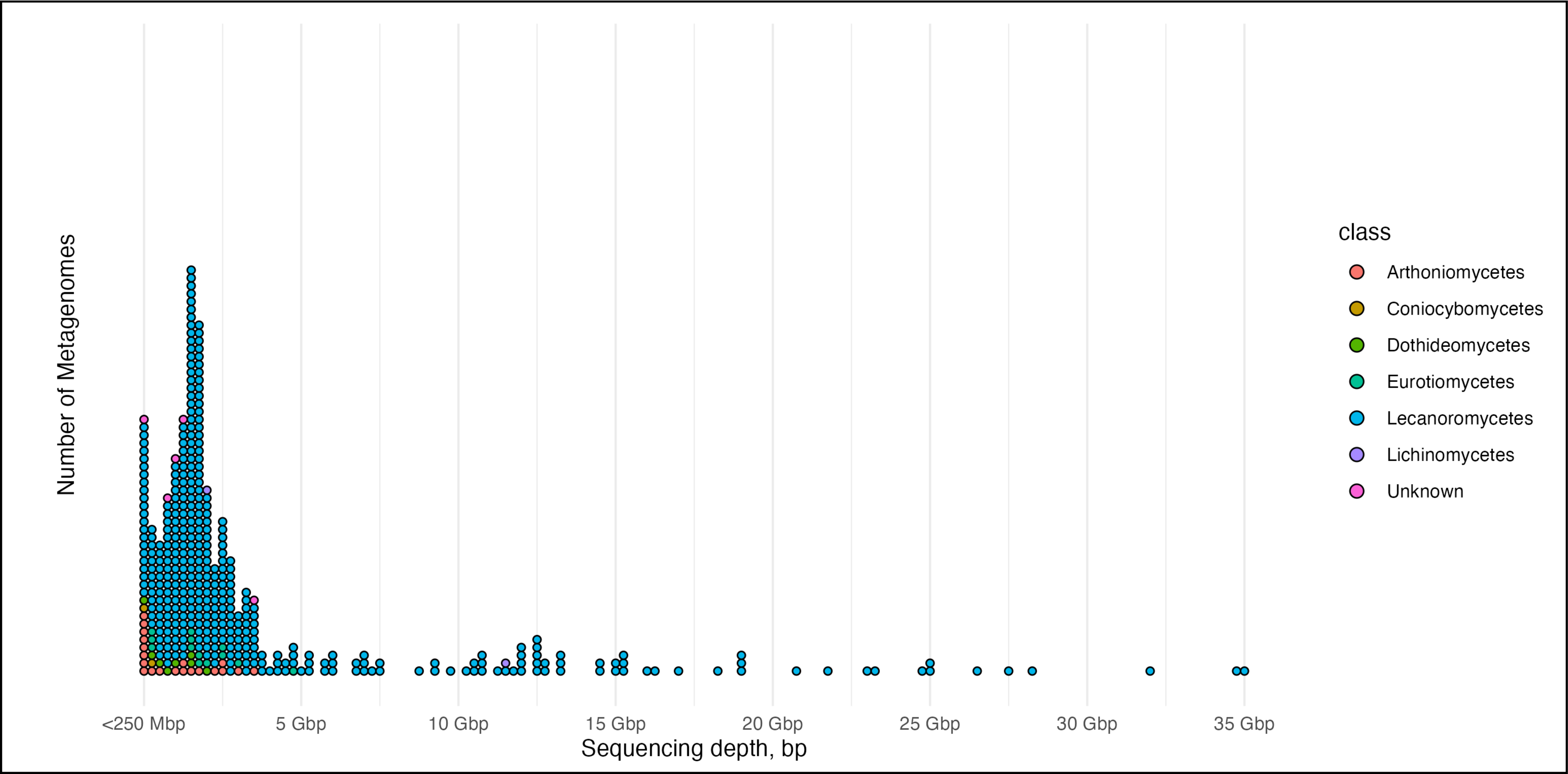

Supplement: S1 Fig — Each metagenome is shown with a dot; the dots are colored according to the taxonomy of the lichen fungal symbiont (LFS). The dots are arranged on the x-axis based on the sequencing depth (bp) and are “stacked” on top of each other. The data underlying this figure can be found in S1 Table. (TIF) [file pbio.3002862.s001.tif]

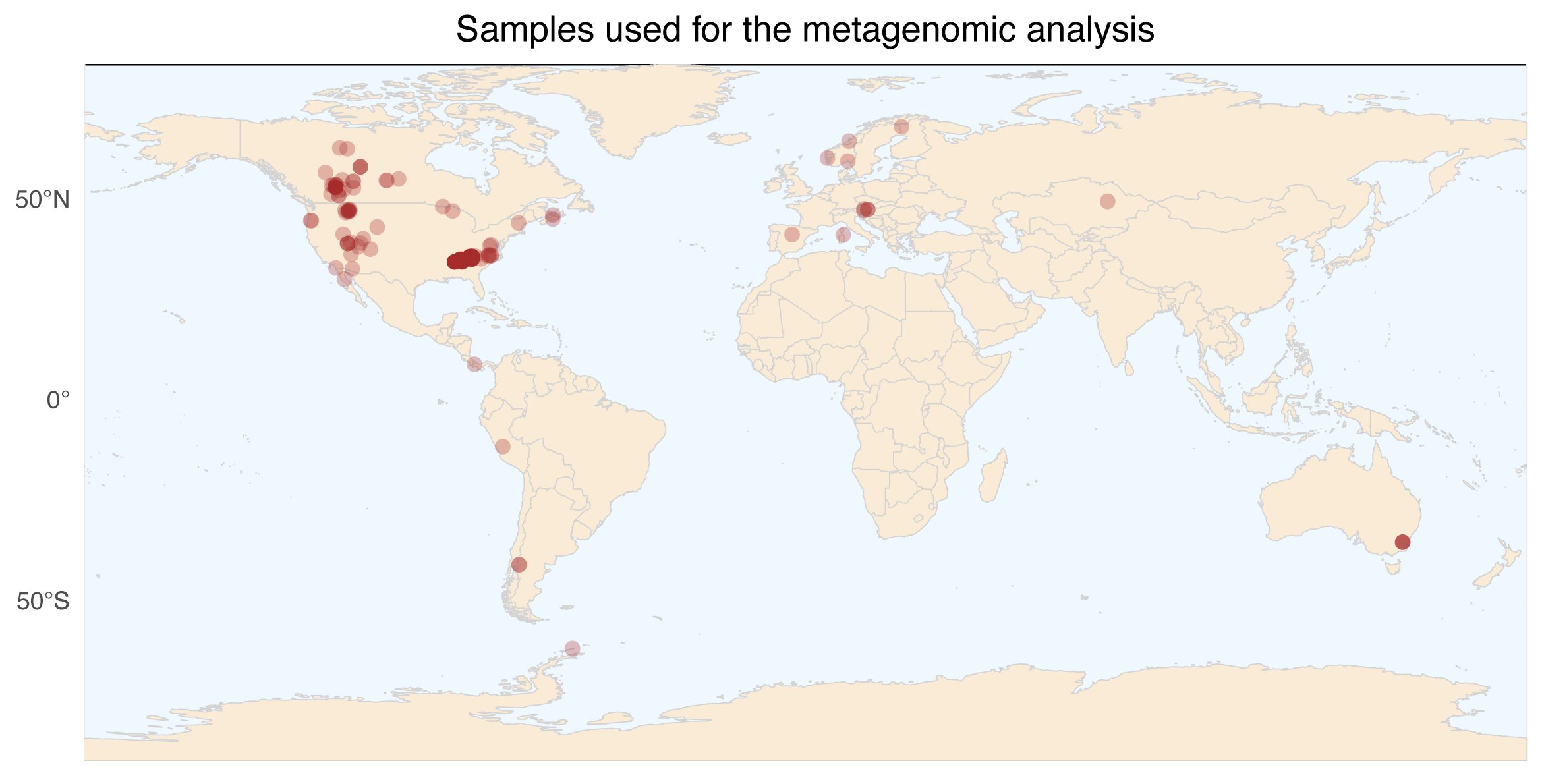

Supplement: S2 Fig — Each dot represents a sample used for metagenomic sequencing. The basemap shapefile was taken from the rnaturalearthdata library (v0.1.0) and belongs to public domain (https://www.naturalearthdata.com/). (TIF) [file pbio.3002862.s002.tif]

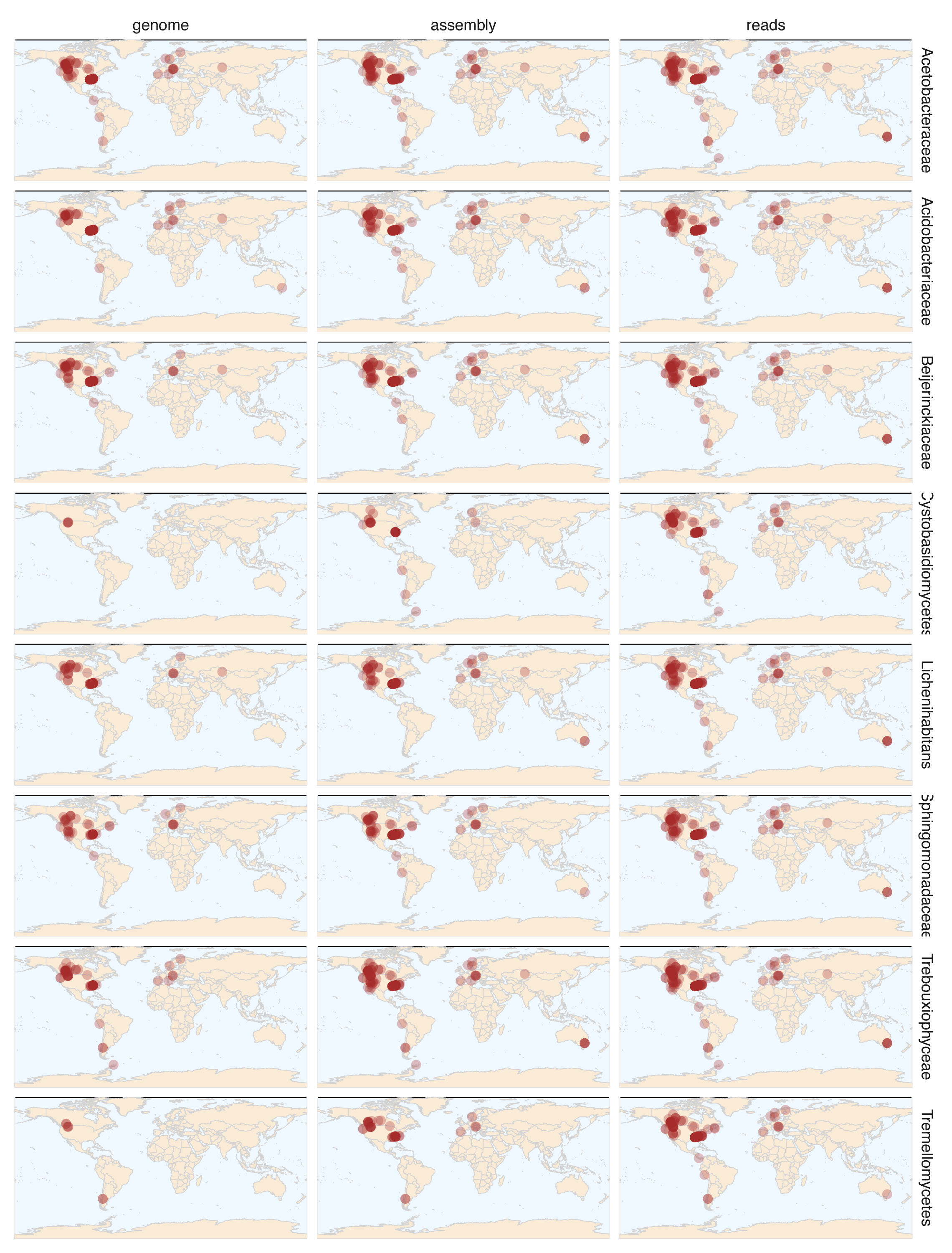

Supplement: S3 Fig — Each dot represents a sample used for metagenomic sequencing. Here are shown data on the 4 most frequent bacterial families, on the genus Lichenihabitans, and on the 3 eukaryotic lineages known to be stably associated with lichens. The basemap shapefile was taken from the rnaturalearthdata library (v0.1.0) and belongs to public domain (https://www.naturalearthdata.com/). (TIF) [file pbio.3002862.s003.tif]

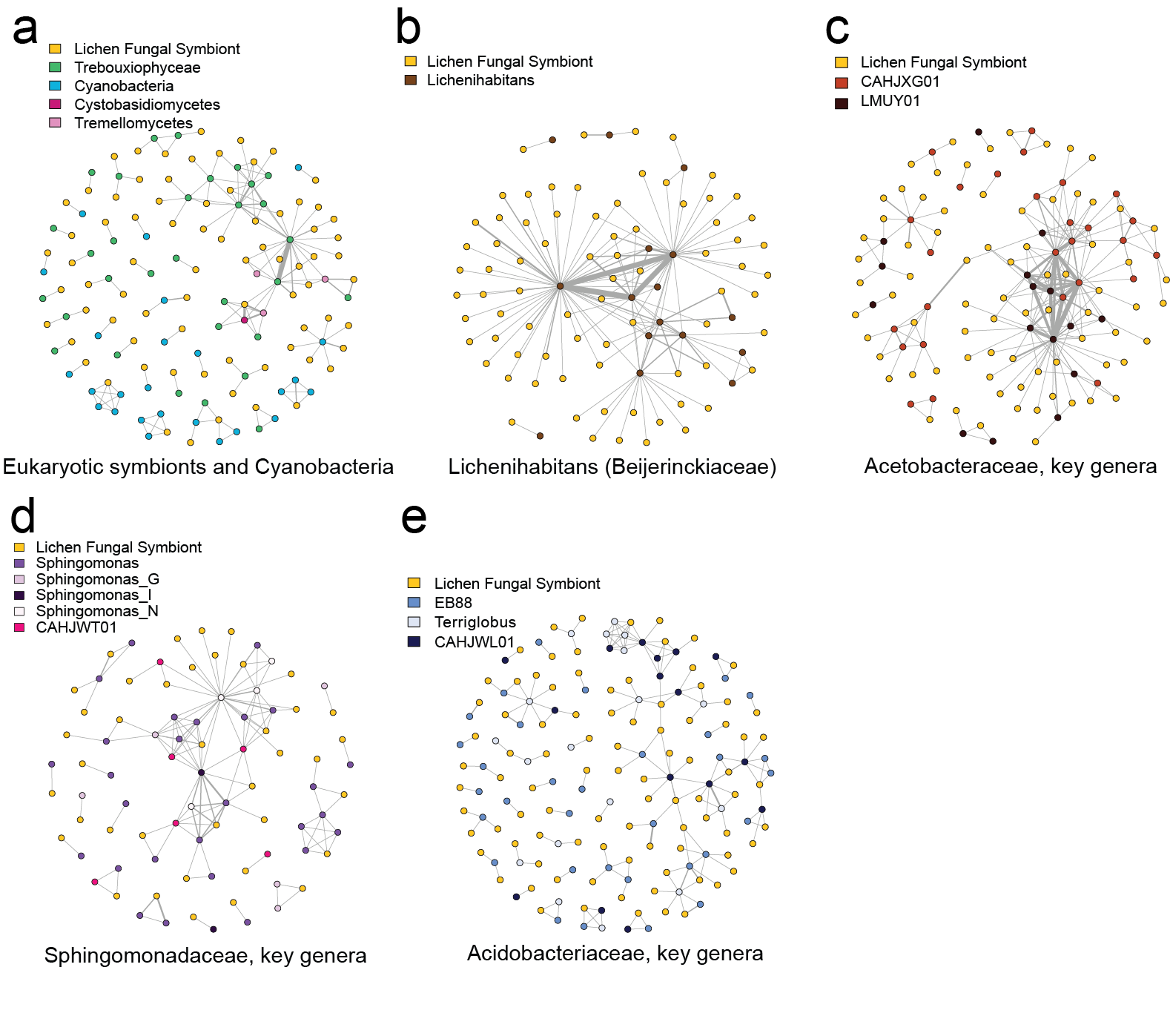

Supplement: S4 Fig — Each node is a MAG, and edges represent the co-occurrence of MAGs within 1 metagenome; the thicker the edge, the more often 2 MAGs co-occur. Nodes are colored based on the taxonomy and function of the symbiont; in each network, yellow nodes represent MAGs of the LFS (lichen fungal symbiont). Only data on metagenomes that yielded an LFS MAG are shown. (A) Co-occurrence of LFSs, other known eukaryotic symbionts, and Cyanobacteria. (B) Co-occurrence of LFSs and Lichenihabitans. (C) Co-occurrence of LFSs and most frequent genera of Acetobacteraceae. (D) Co-occurrence of LFSs and most frequent genera of Sphingomonadaceae. (E) Co-occurrence of LFSs and most frequent genera of Acidobacteriaceae. The data underlying this figure can be found in S1 Data. (TIFF) [file pbio.3002862.s004.tiff]

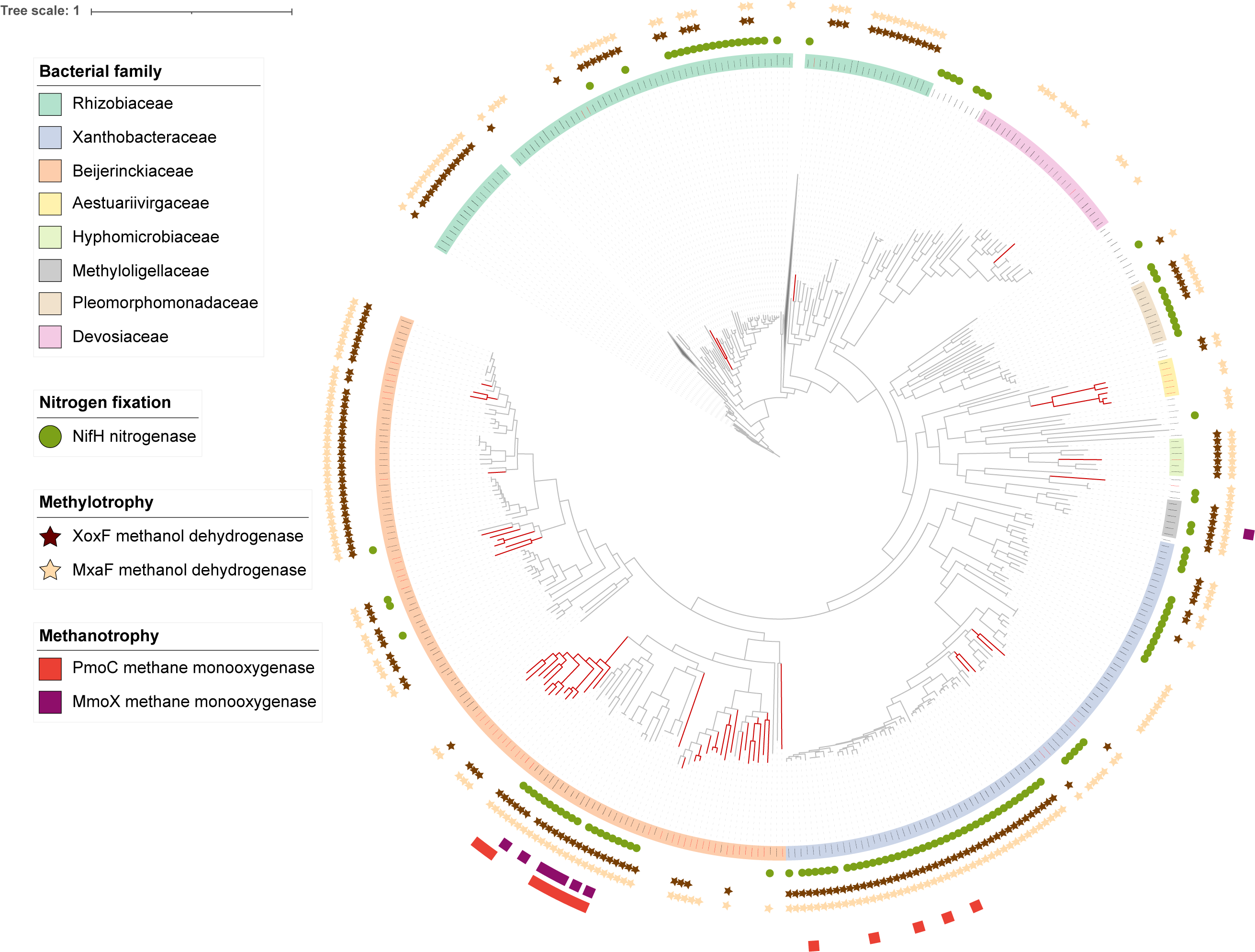

Supplement: S5 Fig — The tree includes published genomes of Hyphomicrobiales and the Hyphomicrobiales MAGs derived from the lichen metagenomes (indicated in red). We generated the alignment of 120 marker genes using GTDB-Tk and calculated the tree using IQ-TREE. The color represents family-level taxonomic assignment. We used tblastn to search the genomes for key genes involved in nitrogen fixation and C1 metabolism. The presence of these genes is indicated with symbols. The full-size version of the tree in the Newick format is available at FigShare (10.6084/m9.figshare.27054937). (TIF) [file pbio.3002862.s005.tif]

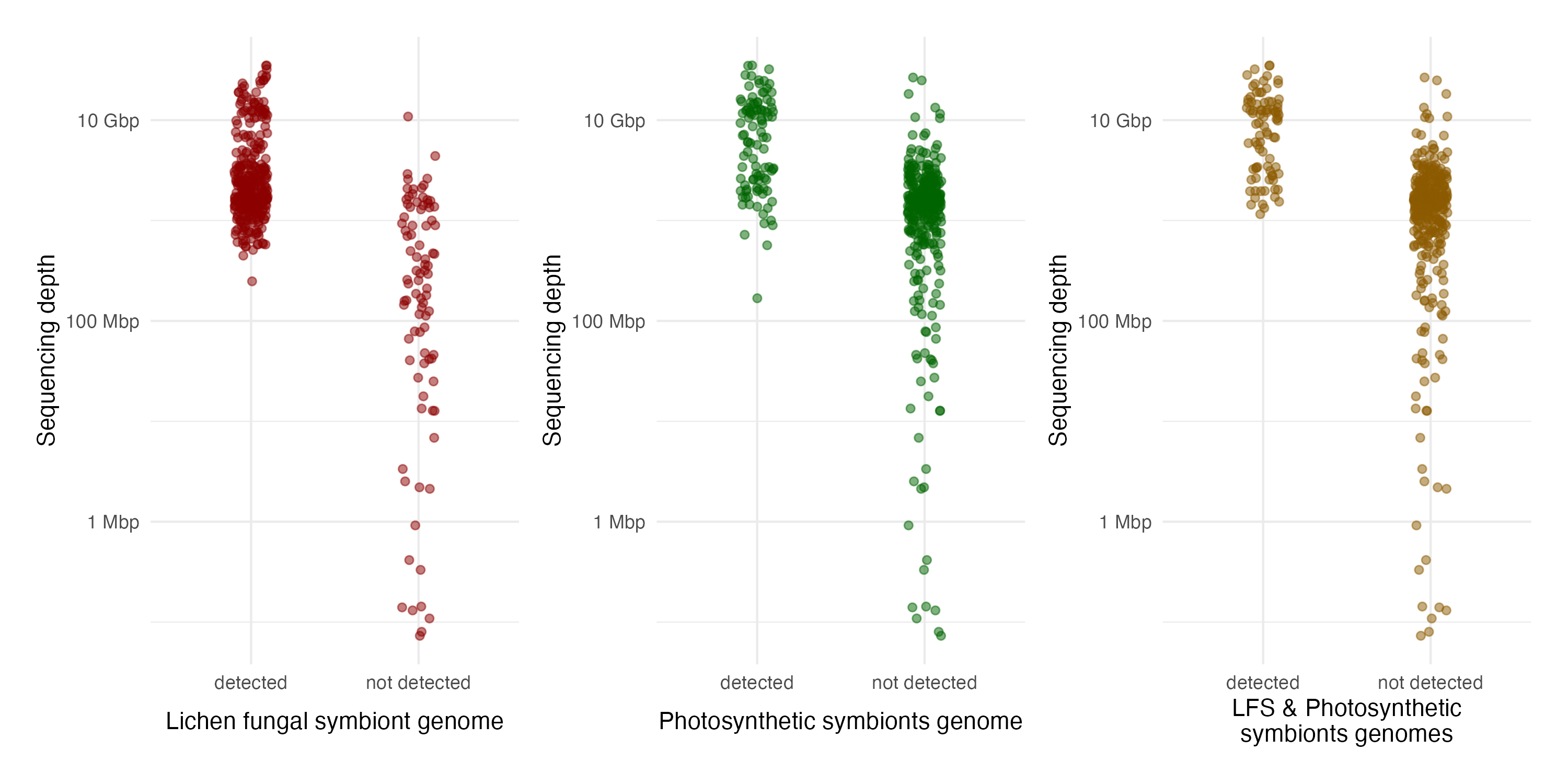

Supplement: S6 Fig — These graphs are based on the pre-dereplication set of MAGs, each dot represents a metagenome and is positioned based on its sequencing depth and on whether it contained MAGs assigned to one or both of the 2 main partners: (A) the LFS (lichen fungal symbiont); (B) the photobiont partner; (C) both the LFS and the photobiont partner. The data underlying this figure can be found in S1 Data. (TIFF) [file pbio.3002862.s006.tiff]

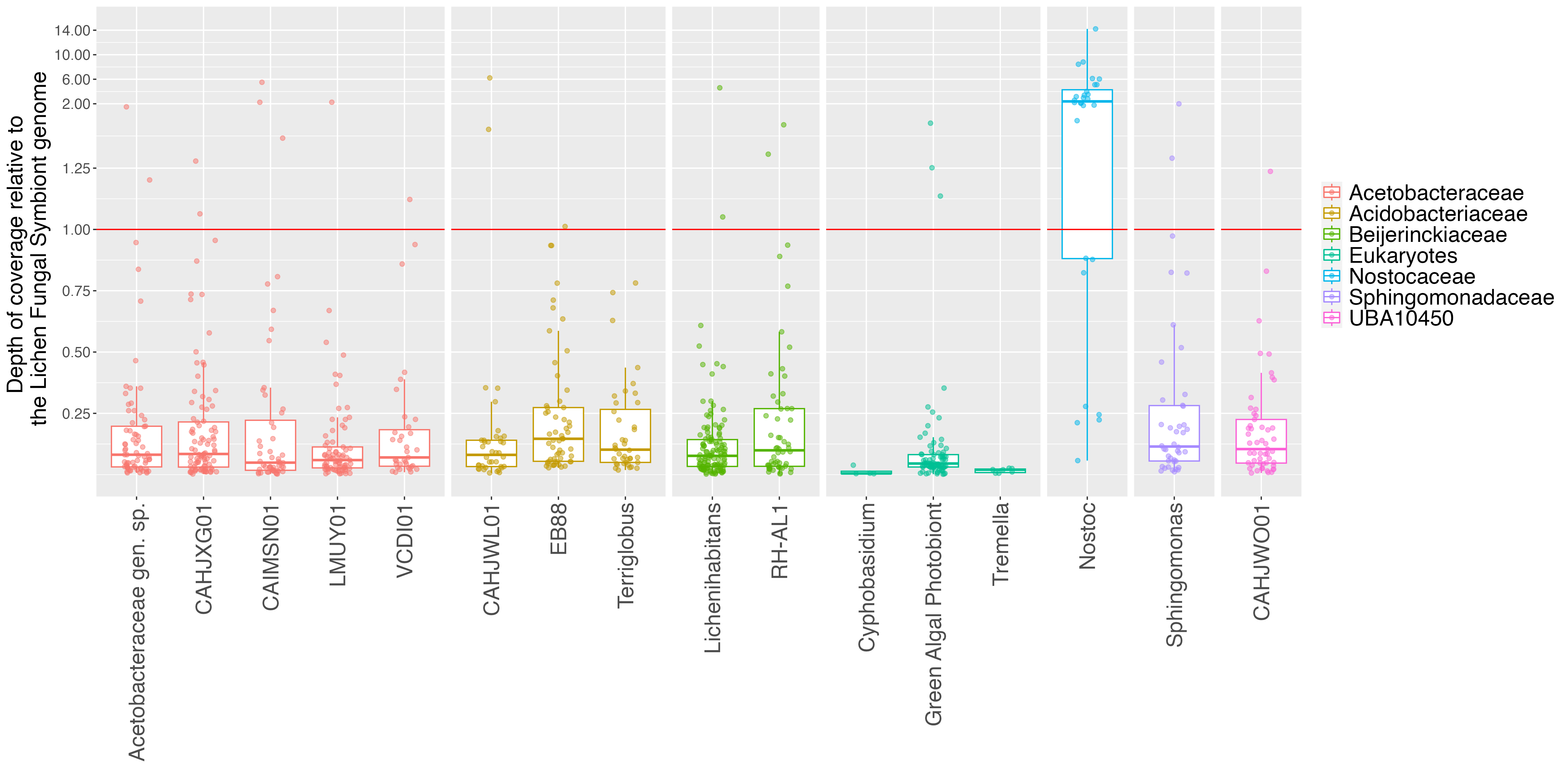

Supplement: S7 Fig — The relative abundances were calculated by dividing the coverage depth of the symbiont MAG by the coverage of the LFS MAG. Here are shown data on the 13 most frequent bacterial genera and the eukaryotes known to be stably associated with lichens. The red line shows 1:1 ratio, where the symbiont is estimated to have the same cellular abundance as the main fungal symbiont. The boxplot elements are defined as: center line, median; box limits, 25th and 75th percentiles; whiskers, 1.5× interquartile range. The data underlying this figure can be found in S1 Data. (TIFF) [file pbio.3002862.s007.tiff]
