# Supplementary material for: Microbial occurrence and symbiont detection in a global sample of lichen metagenomes
Source: PLoS Biol. 2024 Nov 7;22(11):e3002862. doi: 10.1371/journal.pbio.3002862 (PMC11542873; doi:10.1371/journal.pbio.3002862)
Supplement: Attachment — Submitted filename: Responses_to_reviews_PLOS_July_2024.docx [file pbio.3002862.s031.docx]

**Response to Editorial and Reviewers’ comments: Tagirdzhanova et al. (Aug 2024)**

Editorial decision letter:

As previously discussed, reviewer #1 is positive, saying that the paper is now more nuanced and re-framed in a more cautionary manner. Their requests are textual and fairly minor. Reviewer #2 starts positive but thinks that you are too dismissive of the possibility that contamination could have occurred during DNA isolation, and worries about your data being uploaded to GenBank without caveats; s/he also says that you missed an opportunity by not sequencing the substrate from which the lichens were obtained. Reviewer #3 thinks that the you still imply symbiosis too readily and is concerned that you propose to write a perspective piece about the topic. S/he recommends further re-framing the manuscript around issues that affect MAGs, rather than relationships between organisms; the rest of this review is a series of points about how your current manuscript is a halfway house, and how to make it more about the pros/cons of MAG approaches.

Your Appeal email rebutted some of the points raised by reviewers #2 and #3, but also proposed constructive measure to address their remaining concerns. As I understand it, these include a) being completely explicit about having zero direct evidence for symbiosis, b) making a circumstantial but strong case for the likelihood of contamination being negligible, c) clarifying why uploading the MAGs is “industry standard” and should not be controversial or problematic, d) incorporating text about the “open” nature of well accepted lichen symbioses (and why this reduces the onus on analysing substrate metagenomes), and e) including the eukaryotic side of the KEGG/pathway analysis to complement the current bacterial ones presented in Fig 6. To my mind, a reader should not walk away from this paper thinking that you have unequivocally demonstrated a symbiotic role (sensu stricto) for the bacterial components, but that the very useful data have been presented even-handedly and the

circumstantial nature of the evidence for symbiosis made clear.

I should also say that when discussing the Appeal decision, the Academic Editor added the following, which you might find helpful: "One clarification that would help prevent misunderstandings is a clear definition of how the term 'symbiont' is being used. The original definition was just organisms of different species in sustained, close physical contact. But a common use of the term is more specific -- an organism playing some role in the interaction. And an even more specific definition is an organism playing a positive role by enhancing fitness of other participants (i.e. mutualists, not parasites). There is clear evidence for the first meaning, not so clear for the latter meanings."

In light of the reviews, which you will find at the end of this email, we would like to invite you to revise the work to thoroughly address the reviewers' reports.

Thank you for the decision letter and the encouragement to address reviewers’ concerns.

In the revised version, we combine our proposed constructive measure a) (“zero direct evidence for symbiosis”) with the proposal by your Academic Editor to be clear about the definitions of symbiosis; this is a new section in the discussion (“What do our results mean for research on lichen-associated bacteria?”), starting on Line 773 (note we used the line numbering from the “clean” manuscript copy). After much discussion and review, we feel it is unavoidable to acknowledge the surprisingly century-long history of calling bacteria lichen symbionts independent of any of our own work, and that it has never been demonstrated that they are really needed by eukaryotic symbionts (line 785). We discuss under which broad, classical definition of symbiosis we surmise the term ‘symbiosis’ has been previously applied, and could ‘arguably’ (L789) be applied based on co-occurrence alone, in a nod to the AE’s comment. Next, however, we emphasize that no support has been advanced for any lock-and-key function, verbatim (L790):

“our results are insufficient to advance a narrower, function-based case for bacterial symbiosis in lichens”

We suggest that future work could go either way. While on the one hand, the vitamin evidence suggests a plausible scenario under which metabolic complementarity could exist between bacteria and e.g. algal-free fungi, we also acknowledge that “It is also possible that biotin-prototrophic bacteria occur in such small amounts that their contribution to eukaryotic metabolism is physiologically negligible” (L797 ff.).

Item b) (“making a circumstantial but strong case for the likelihood of contamination being negligible”) we have addressed by adding two new sections. The first, starting on line 657, outlines what we knew the overall bacterial dataset to contain at the outset, the decisions we made to select a subset with reduced contamination likelihood, and the constraints we assume this places on inference (e.g. ability to infer presence, but not absence). The second paragraph, starting on line 826, explicitly addresses the non-trivial issues around separating ‘lichen-relevant’ from contaminant fractions and the pros, cons and untested assumptions around surface treatment, twinning with environmental samples, and PCR-based secondary screening.

Item c) (“clarifying why uploading the MAGs is “industry standard” and should not be controversial or problematic”) we feel is best addressed here, as opposed to in the manuscript. As we address in response to reviewer #2, the standards implemented in our project are consistent with those of other major projects, such as metagenome sequencing of the mouse gut (Beresford-Jones et al. 2022, *Cell Host & Microbe*, <https://doi.org/10.1016/j.chom.2021.12.003>) the catalog of the human gut microbiome, involving part of our team (Almeida et al. 2020, *Nature Biotechnology* <https://doi.org/10.1038/s41587-020-0603-3>), and in the cow rumen (Stewart et al. 2019, *Nature Biotechnology* https://doi.org/10.1038/s41587-019-0202-3). The raw sequence data from lichens are in any case in the SRA and free to mine by anyone. The MAGs generated from these analyses, however, do not flow into Genbank but are submitted to the dedicated MAG layers in ENA, where they are associated with quality (completeness and contamination) metrics but are agnostic with respect to their status in the organism.

Item d) (“incorporating text about the “open” nature of well accepted lichen symbioses” [and why this reduces the onus on analysing substrate metagenomes]) we have included in a revised section near the end of the discussion, starting at line 834.

Item e) (“including the eukaryotic side of the KEGG/pathway analysis to complement the current bacterial ones presented in Fig 6”) has been added as its own section in the methods (line 1022), results (lines 482 to 555, including the new figures 6 and 7) and discussion (starting on line 745), as well as a brief shout-out in the abstract.

As to the final point (“a reader should not walk away from this paper thinking that you have unequivocally demonstrated a symbiotic role”) — we hope that this is the case, and that the reader comes away with an appreciation for what can and cannot be extracted from lichen metagenomes and how newly extracted data inform the century-long study of lichen-associated bacteria.

We think the totality of our results — the reported limitations with eukaryotes, the six main points bulleted from line 692 onwards and the newly highlighted arena of cofactor metabolism, added for this revision — present a major set of new data that have changed how we and our colleagues see metagenomes as resources and injected a large amount of new data on bacteria in and on lichens.

REVIEWERS' COMMENTS:

Reviewer #1:

Tagirdzhanova et al.

Microbial occurrence and symbiont detection in lichen metagenomes

This manuscript is a resubmission of a manuscript previously reviewed at Nature and Cell Genomics. I was a previous reviewer and have read the extensively revised manuscript and the complex set of reviews and author responses from the earlier submissions. The authors have made a major effort to address my previous comments and, I believe, those of the other reviewers. I recommend that the paper be accepted with minor revision.

Specific comments:

-Most generally, I want to highlight what I see as the most important element of the revised manuscript. It has a revised thrust and presents an important set of take-home messages and cautionary tales for lichen researchers, and metagenomicists in general. I won't rehash them here, but basically they are as follows: given what we know about the biology of lichens, there are things we can trust and things we cannot trust about MAG data. We now know enough to know that there are a variety of factors contribute to the extent to which key players in lichens are recovered in MAGs. Sometimes they are not recovered at all, despite knowing from other lines of inquiry that they are there (e.g., basidiomycetes). This is sobering and important for the community to take on board.

Thanks for the positive feedback.

-Second, my opinion is that the authors have taken a suitably nuanced perspective of the concept of symbiosis and acknowledge the myriad uncertainties. The Discussion section on page 25-26 stands out in this regard, as does the "Limits to inference" section that immediately follows, which brings the question of 'surface sterilization' out into the open. The authors seem to be asking the question: do lichens [whatever that means] have a microbiome? I think this is an important question to ask and debate amongst researchers in the field. Side-stepping use of the term 'core' doesn't make the underlying issues go away.

Thank you. In the present, revised version we have moved this content into an expanded section of the discussion under the subheader “high frequency bacteria as the focus of detailed analysis”.

-The introduction is truly excellent - it provides an authoritative overview of the history and current state of affairs in modern lichen research. It reads a bit like a thesis intro (maybe it is); certainly the extensive referencing (well over 100 citations) is a strength of the paper, to the extent that it aligns with the constraints of PLOS Biology.

Thank you.

-The 24 newly generated metagenomes are basically an afterthought in the current paper. Yes, they are embedded within a much larger study and analytical / interpretive framework, but readers will be interested in where they came from and how they were generated (provided on page 19, the very last section of the Results). Space permitting, draw more attention to it in the abstract (line 31) and consider moving this further up the results. I see why it is placed where it is - but maybe a nod to these data and their significance earlier would be helpful too.

We have added a brief explanation why we included these metagenomes — as we put the dataset together and examined the role sequencing depth plays, it became clear that high sequencing depth metagenomes were underrepresented, and we had some unpublished material that could be used to augment this part of the dataset.

-I am not sure why the authors chose to embed the figure legends but not the figures into the main text. This was not helpful.

Apologies — we were/are trying to follow the author guidelines (screen shot):

-Line 127 - "We began by removing identical metagenomes that have been published twice in the sequence databases (n=43; S3 Table)". Meaning unclear based on main text alone. My brain instantly asked: do they mean literally the same dataset or independently sequenced metagenomes from the same location / environment by different labs? The former makes sense to remove, the latter would seem to be a useful control for experimental and bioinformatic procedures. This becomes clear from the supp info line 1381/82, i.e., they are literally the same data submitted twice to GenBank with different accession numbers, which of course is unfortunate. I suggest the authors make this clear in the main text as well.

Done. We have added, in parentheses, that these are not biological replicates.

-For much of the intro, the LFS acronym is used in the singular (e.g., "the lichen fungal symbiont (LFS),..."). This of course becomes clear that they are indeed exploring and discussing the presence of basidiomycetes as well. I suggest working in acknowledgement of the presence of multiple types of fungi earlier on. Why? Even non-specialists / lichen enthusiasts will be aware of the high-profile Science paper describing multiple fungal partners. The way the intro reads at first it almost sounds as though the authors are back-tracking on that observation, which they are not.

Our use of “lichen fungal symbiont” is consistent with our recent papers (e.g. *Nature Communications* 13: 2634; *New Phytologist* 234: 1566-1582) in which we treat the main fungal symbiont as LFS and e.g. recently detected basidiomycete yeasts as additional constitutive symbionts in some lichen groups. None of this is inconsistent with the *Science* paper, in which we clearly stated that secondary fungi had been detected in about 75% of parmelioid lichens (a subset of all lichens). However, it might appear to be in contrast to some of the media reporting on that paper, which made it sound like all lichens are symbioses of two fungal symbionts, and that we know that lichens require secondary fungi to form (we do not know this). We have again clearly stated our position on this e.g. in a recent book on lichen natural history (Lücking & Spribille, *Lives of Lichens, A Natural History*, Princeton, 2024: page 58).

Reviewer #2:

This manuscript presents an extensive analysis of lichen metagenomes aimed at elucidating the microbial composition and detecting symbiotic relationships within lichen ecosystems. The authors have reassembled and analyzed over 400 publicly available metagenomes, generating metagenome-assembled genomes (MAGs), constructing phylogenomic trees, and mapping MAG occurrence. The study provides valuable insights into the prevalence and diversity of bacterial, algal, and fungal constituents across a wide array of lichen species.

Thank you for the positive feedback.

Before the submission to PLoS Biology, the manuscript was submitted to the journals Nature and Cell. Reviews from these earlier submissions were attached to this submission.

The authors of the manuscript have made a significant change in the latest version by reducing the emphasis on describing a "core" and "global" set of microbial lichen symbionts. This change aligns with the recommendation of many reviewers. Although the authors' responses to the reviews suggest that they are not entirely convinced that their data does not allow such an evaluation, the changes are still appreciated. The new submission compares various methods to extract lichen metagenomes and presents the results more holistically by focusing on the total pool of genomes recoverable from the lichen data.

Reviewers have pointed out that there is contamination in both the public dataset and the data produced by the authors. I disagree with the dismissive response from the authors. The contamination could have occurred during DNA isolation, before the barcoding. Although the authors provide an unlikely biological explanation, I am surprised they decided to ignore this contamination. I assume they still plan to upload this data without commenting on it to Genbank, which could cause problems for colleagues who want to work with the data. Furthermore, I am concerned that these samples may be representative of other inconsistencies in their data.

We apologize if our response came across as dismissive; we have always had it as our priority to execute a series of control and filtering steps to minimize the likelihood that the subsets of data we analyze are contaminants. In the revision, we have made an effort to better explain the sequence of decisions made about which parts of the bacterial data to analyze in depth, which ended up being a quite narrow and conservatively delimited set. Specifically, we have added three new sections/comments to the discussion.

- One discusses the known attributes and limitations of the bacterial data from the outset (line 657) and how this led to narrowing the analyzed data and narrowing the inferences we acknowledge we can make from them.
- The other new section (starting line 826) incorporates text that was previously in the “limits to inference” section, in which we address how future work could consider the possible effects of ‘surface sterilization’ on lichen microbial composition and cell abundance; and what we stand to learn from studying the occurrence in surrounding environments of lichen-associated microbes, noting that even canonical lichen symbionts such as eukaryotic photobionts are already known to be widespread in such environments.
- Finally, we note that the major bacterial groups we analyze both corroborate the results obtained from independent studies of lichens and exhibit little to no overlap with the reported composition of bacterial contaminations in lab kits (line 820).

Regarding uploading the data to Genbank: we note that the raw sequence data are in any case in the SRA and free to mine by anyone. As to MAGs, we have submitted our MAGs to the dedicated MAG layers in ENA, where they do not flow into GenBank. The quality (completeness and contamination) of these MAGs is recorded in the submission, and the MAGs are linked with the short-read sequence record. Each MAG record is agnostic with respect to its status in the organisms from which it was sampled.

Additionally, I believe there was a missed opportunity with the data they produced themselves. They could have addressed many of the criticisms raised by the reviewers by conducting further testing. For instance, they could have sequenced the substrate in addition to the lichen symbiosis to determine how many of the bacteria identified by sequencing were associated with the lichen and how many were sequenced by chance. Furthermore, they could have treated the same sample differently to test if they were correct in their speculation that irrespective of the treatment, the sequencing results would have remained the same.

The added control the reviewer proposes assumes that organisms occurring as lichen symbionts are unique from those in the surrounding environment. However, we know that this is not the case for the algal symbiont (see e.g. the review of Vesela et al. 2024, now cited in the text, line 840) and we cannot rule out that this is not the case for any other microbe, or that it even matters biologically. We knew this going into this study and do not and have not viewed this as a limitation of the metagenome data. In an added section, we now point this out and state that exploration of the occurrence of lichen-associated microbes in their surrounding environments is an area beyond the scope of the study that could be insightful with respect to their biology, but that we simply do not know enough about lichen biology to say that occurrence outside of a lichen rules out a microbe from participation in the lichen system.

The manuscript uses a metagenomic methodology to assess lichen symbioses, which is a valuable addition to the field. However, I have concerns regarding the inconsistencies in the presented data and the missed opportunities for further investigation. These factors cast doubt on the manuscript's suitability for publication in PLoS Biology.

The acknowledgement of the metagenomic method is appreciated here, but we note that Reviewer 2 appears to emphasize mechanical sample treatment, handling and pairing with environmental samples as the preferred method(s) for narrowing the sampled microbe pool to assumed lichen-specific microbes. However, we view the extraction of informative signal on lineage occurrence from our data as a bioinformatics problem, much like MAG binning itself, in which signal can be separated from noise by leveraging data attributes without a reference. We do not consider the absence of sample treatment or twinning with environmental samples to preclude the bioinformatics-driven, sequential subsetting and analysis of the available data which we performed; and state that we simply do not know enough about lichen biology to know that fractions subtracted by surface treatment or environmental composition triangulation are not lichen-relevant (line 826 ff.).

Reviewer #3:

Dear Authors,

I have carefully reviewed the manuscript and the comments of other referees. Although I admire the effort behind putting together a manuscript, I have several major concerns, which prevent me from recommending its publication in its current form. Some of my major concerns align with those of the referees who reviewed the manuscript before.

Firstly, while the manuscript has shown improvement in certain areas, such as refraining from referring to the identified bacteria as core, the central narrative remains somewhat vague. Throughout the manuscript, you imply that bacteria are symbionts, yet refrain from stating this directly due to the lack of sufficient evidence to support the claim, as advised by most referees.

We have consolidated our reply to the reviewer’s concern about the implication of symbiosis into a single response, see below (the section starting after points 2–5).

Specifically, while the referees have expressed concerns about the adequacy of evidence supporting this classification, you argue that the presence of bacteria, including on the unsterilised surface justifies them to be part of the biofilm and you also propose that bacterial gene pathways are beneficial and functionally relevant for fungi based on their mere presence in bacteria. However, the sole presence of microorganisms on an unsterilized surface does not necessarily imply a symbiotic relationship. Do we consider viruses and contaminants from the environment, such as dust and sand particles, as a part of biofilm and tag them as symbionts? No. Then why bacteria. Simply because bacteria have the pathways that could potentially be beneficial for fungus does not mean the identified bacteria are symbiont. Do closely related non-symbiont bacteria lack these pathways?

We approach this as microbiologists and consider bacteria, as osmotrophs, to be potential contributors to any system until proven otherwise. This is especially true of a lichen, where even agreed-upon symbionts are microbes and goods and services are exchanged across an extracellular matrix that cannot be sterilized like a plant leaf. That being said, we agree that the use of the term “symbiont” has been used without sufficient definition in lichen biology. We have added two sections that hopefully will clarify our interpretation of the data:

- First, we have taken the advice of the Academic Editor by adding a section at the end of the discussion, in which we try to clearly state a) what has been claimed before and b) what we can and cannot say with our data under the differing definitions of symbiosis.
- Second, we have added a new section on what we consider the non-trivial problem of demarcating what is ‘lichen-relevant’ and contaminant and how different assumptions may affect where this line is drawn.

Until sufficient evidence is generated, I would suggest to refrain from making such claims.

Furthermore, your reliance on publishing a perspective to support your argument may indicate a lack of openness to alternative viewpoints, which undermines the purpose of peer review. You have faint to no support for their idea of bacteria being symbionts but you argue that you will publish a perspective on this. So you will increase the support for your claim by publishing opinions and perspectives. We should be careful about supporting such approach that an idea/perspective is suggested as a hypothesis but becomes a "suggestion" and eventually a fact. A perspective therefore would not give more support to bacteria being symbionts.

The reviewer is referring to a statement we made in response to Referee #3 in the Nature review. Specifically, we wrote:

“the debate about what is a symbiont or not, in lichens, is being had largely in

absence of a conceptual framework for what the expectations are in terms of population

structure and thallus integration of symbionts, a topic that is beyond the scope of the

present paper, but which we plan to address in an upcoming perspectives piece. Exhibit

A of this problem is that lichenologists have no problem (now) accepting green algae as

lichen symbionts, despite the fact that in many documented cases, no single species

consistently consorts with one fungus; it is a rotating cast of players, or even more than

one at a time. When a similar pattern is detected in yeasts, as was done by Mark et al.,

this is considered somehow disqualifying for the title of ‘symbiont’.

In the present paper, we avoid overuse of the term since we feel a review and

perspectives piece is needed before we further stir up the semantic hornet’s nest in our

narrow subfield.”

To be clear, we had and have no intent to use this future perspective piece as a vehicle to increase support for any claim. Instead, we hoped it would help facilitate a conversation about the attributes of microbes considered to be symbionts in lichens, based on what we know about how currently agreed-upon symbionts interact, and discuss whether rigid categories like “symbiont” are even useful given what we are learning about the context-dependency of microbial interactions (similar to the debate around microbial pathogens: Casadevall & Pirofski 2014, *Nature*, https://www.nature.com/articles/516165a).

For the present paper, the Academic Editor has helpfully suggested that "One clarification that would help prevent misunderstandings is a clear definition of how the term 'symbiont' is being used. The original definition was just organisms of different species in sustained, close physical contact. But a common use of the term is more specific -- an organism playing some role in the interaction. And an even more specific definition is an organism playing a positive role by enhancing fitness of other participants (i.e. mutualists, not parasites). There is clear evidence for the first meaning, not so clear for the latter meanings."

We have now implemented this in the discussion (line 774 onwards) and point out that there is no support for the lichen system needing bacteria, neither previous to our study (line 784: “to our knowledge no studies have shown any metabolic function that locks the now-canonical fungal and photobiont symbionts into a dependency on bacteria, or *vice versa*”) nor after our analysis of the newly available MAGs (line 790: “our results are insufficient to advance a narrower, function-based case for bacterial symbiosis in lichens”).

Moreover, I suggest refraining from name-calling, such as "Duke school," and instead showing respect for alternative hypotheses. I understand the frustration given the work put into a manuscript. However, given the insufficient evidence to support a strong symbiotic relationship, it's important to acknowledge and address the specific concerns raised by the referees.

We do acknowledge the legitimate concerns and thank you for the effort you put into expressing them. No offense was meant with the term “Duke school” and we apologize if anybody was offended.

As the central idea of the manuscript is not supported by the evidence, I would recommend major restructuring.

Best regards.

Major concerns:

1) The manuscript introduction appears to lack a clear direction, with an imbalance evident in all the sections. For instance, given the title of the manuscript "Microbial occurrence and symbiont detection in lichen metagenomes" and the main knowledge gap being "overall content and limitations of these metagenomes have not been assessed," I would expect the introduction to center around issues of MAGs and risks of bacterial read contamination. Instead, there is an undue emphasis on explaining the relationship between fungi and algae. This suggests that the manuscript may have undergone several rewrites, leading to a loss of focus and coherence in conveying the central story.

Overall manuscript direction: a core outcome of the metagenome analysis is that metagenomes are both data-rich and can be mined for information never before seen, and also can be limited by constraints on MAG assembly. We are well aware that this is not a simple, linear message, but we feel it is the most accurate way of presenting what we found.

Introduction: we carefully considered what aspects of lichen biology a reader not familiar with lichens might require to frame and understand the use of metagenomics on the system, and this informed how we wrote the introduction. We note that Reviewer #1 found the introduction “truly excellent”. After consulting with several other focus group readers on the introduction content, we have opted not to substantively change it.

Points 2 through 5 share a common theme, highlighted in yellow, and we have consolidated our reply in a single response, below.

2) My second major concern aligns with one of the reviewers, regarding the use of MAGs to identify lichen-associated bacteria. This also explains the use of convoluted terminology between the abstract and introduction to state the main aim of the paper. While the manuscript progresses, the authors shift from "overall content and limitations of MAGs have not been assessed" to implying consistently that bacteria are symbionts but without ever stating it directly. They keep referring to the studies that investigated the possibility that bacteria are symbionts.

3) Between the two possible directions of the manuscript (bacteria in MAG or bacteria as lichen symbionts), based on the data, I would recommend opting for the first one as hinted in the abstract. For the second option, 1) the data is not apt, and 2) even if the presence is established, there is not enough or any evidence for stating that bacteria are symbionts (see detailed comments below).

4) My fourth but actually one of the biggest concerns is that despite various reviewers in two rounds of reviews, the authors are not keeping an open mind to the fact that several unrelated reviewers, for one reason or another, but all very strong, advise the authors that they do not have evidence to even suggest that bacteria are symbiotic to lichen associations or lichens are composed of fungus, algae, and bacteria. It seems that the authors got fixed on the idea of proving bacteria are symbiotic in lichens, and they are looking at everything through that lens, completely ignoring the concerns of all the reviewers.

5) Misleading idea. Starting with bacteria in MAGs to slowly transitioning to bacteria are symbionts is conveying that authors want to make a claim without evidence but being stopped by the reviewers' refrain to state it directly but keep implying it through the manuscript. I would strongly recommend against it. Please define an aim, state a knowledge gap, address it, and REFRAIN not only from stating but also implying the conclusions not supported by evidence.

Response: a common theme of points 2 through 5 is that we imply that bacteria are symbionts (highlighted in yellow). In fairness to the reviewer, the original BioRxiv version of this manuscript liberally used the term symbiont without clearly stating a definition of symbiosis; and they are correct that in subsequent revisions we reduced the use of the term while referring to the literature on the study of “bacteriobionts”, which overlap with many of the lineages we detected; and they are also correct that we were not explicit on our own interpretation of whether these bacteria are symbionts or not, and under which definition.

The Academic Editor helpfully suggested a possible solution: that we provide clarity by being explicit about which definition of symbiosis we use (see editorial decision letter, above). We have taken this advice by adding a section at the end of the discussion, in which we try to clearly state a) different definitions of symbiosis (line 774 to 782), b) what has been claimed before (line 773, 776) and b) what we can and cannot say with our data under the differing definitions of symbiosis (line 788 onwards).

Specific comments

1) The manuscript introduction appears to be lacking a clear direction, with an imbalance evident in all the sections.

For instance given the title of the manuscript "Microbial occurrence and symbiont detection in lichen metagenomes", and the main knowledge gap being "overall content and limitations of these metagenomes have not been assessed" I would expect the introduction centers around issues of MAGs and risks of bacterial read contamination.

Instead, there is an undue emphasis on explaining the relationship between fungi and algae. This suggests that the manuscript may have undergone several rewrites, leading to a loss of focus and coherence in conveying the central story.

This appears to recapitulate point #1 from “major concerns verbatim; see reply there.

As the introduction flows the primary focus of the paper, i.e., bacteria in MAG (if we read the title and abstract), shifts to bacteria as lichen symbionts. See below

Abstract: In lichen research, metagenomes are increasingly being used for evaluating symbiont composition and metabolic potential, but the overall content and limitations of these metagenomes have not been assessed. We reassembled over 400 publicly available metagenomes, generated metagenome-assembled genomes (MAGs), constructed phylogenomic trees and mapped MAG occurrence and frequency across the dataset.

Introduction: (lines 86-89) However, though hundreds of bacterial strains have been detected, overall few lichen symbioses have been censused for their bacterial composition, with most work focused on the model symbiosis involving the ascomycete fungus Lobaria pulmonaria (reviewed by Grimm et al. [13]).

Discussion:

lines 446-450

Our analyses of lichen metagenomes yielded two contrasting sets of results. On the one hand, our data provide evidence that bacteria that have until now been studied as potential lichen symbionts in only a couple dozen lichens in fact occur in or on hundreds of lichens, and by extracting over 600 MAGs we increase the amount of available genomic data by orders of magnitude.

We do not accept the premise of the reviewer’s argument that we are surreptitiously trying to build the case for any group of bacteria as symbionts. They are already referred to as symbionts in many places in the literature, rightly or wrongly (and we now explicitly address this in the section on definitions, as per Academic Editor recommendation; line 776) and we are presenting the MAGs as a resource to scrutinize claims about their metabolic involvement in lichens. We unambiguously state that we do not currently have evidence to support or rule out that bacteria are required, function-performing symbionts.

Results:

If the paper is about overall content and limitations of these MAGs

What is the logic of analyzing the bacterial pathways and genes and their functional categories? In that case authors analyze the pathways present in bacteria from different labs when the samples were prepared

The reviewer has presented no evidence that the annotations we are presenting are from contaminant bacteria, but we present multiple lines of evidence why we think they are not.

If the paper analyzes the possibility of bacteria being symbionts

1) The dataset is not suitable. In short most lichen genomes are generated to obtain the fungus and hence may not implement appropriate sample preparation protocol and environment.

2) As the data is generated by several different labs, the amount of bacteria in the samples is uncomparable.

3) Even if we ignore these points and consider the dataset is suitable for the study, the study only shows bacteria are present in the thalli but if the pathways that bacteria contain benefit the fungus is anything but pure supposition, especially because some of these bacteria may also be free living or associated with other organisms. In that case one must prove that these pathways exist entirely in bacteria symbiotic with lichens to say that these pathways are relevant for fungi and hence bacteria are symbionts.

.

- Just because we felt/feel it necessary to acknowledge other people’s work on bacteria as potential lichen symbionts and used this language in its broad sense in earlier versions does not mean this paper has or ever had the singular goal of deciding whether some bacteria can be crowned as symbionts.
- As to what the data are suitable for, we have now added a section about data set attributes that frame or limit inferences and what we consider can and cannot be said about the data (text leading up to line 682).
- As to the second point, we do not make quantitative comparisons — the only comparisons we make relate to relative coverage or occurrence frequency, and in the current revision we explicitly address the difference between reporting minimum occurrence frequency (which we do) and asserting absence (which we do not do).
- With respect to the third point, if we understand this correctly the reviewer is suggesting we should not explore the newly available annotated MAGs of high frequency bacteria from the same taxonomic groups widely under study against claims that have been made in published literature. If this is what they mean, we disagree.

I would suggest to define the aim of the manuscript clearly it can either be :

Bacterial contamination in lichenized fungal MAGs; As evident from the abstract lines 28-32

In lichen research, metagenomes are increasingly being used for evaluating symbiont composition and metabolic potential, but the overall content and limitations of these metagenomes have not been assessed. We reassembled over 400 publicly available metagenomes, generated metagenome-assembled genomes (MAGs), constructed phylogenomic trees and mapped MAG occurrence and frequency across the dataset."

Or

Screening lichens for the bacterial composition/symbionts; As implied in the introduction lines 86-89

However, though hundreds of bacterial strains have been detected, overall few lichen symbioses have been censused for their bacterial composition, with most work focused on the model symbiosis involving the ascomycete fungus Lobaria pulmonaria (reviewed by Grimm et al. [13]).

But not implying bacteria as symbionts in either of the above

We do not agree that the aim of the manuscript has to be defined under either of the two headings offered by the reviewer. We have presented the data filtering steps and assumptions that led us to analyze the metagenomes we did, as well as the bacterial MAGs we selected for further analysis, and we believe it is in the common interests of our discipline to make these data available to the community to inform a balanced analysis of microbial interactions and the use of metagenomes in this system. We did not and do not have a desired or preordained outcome.

Introduction contains details and knowledge gaps which have nothing to do with this title or abstract of the article

for instance:

73-76 Although much about the biology of the disparate photobiont lineages remains unclear, especially with respect to what they receive from the fungus, their population structuring vis-a-vis their LFS partners is consistent with the definition of an open symbiosis [20].

Another example: No consensus exists however on how many other symbionts might be involved, and since most other associated microbes lack visible chlorophyll, their detection is not trivial. Researchers as early as Maria Cengia Sambo in the 1920s cultured additional bacteria from lichens, leading to the speculation that lichens could be 'polysymbioses' of more than two partners.

This is an example of slowly shifting focus from bacteria in MAG (implying that bacteria are symbionts?)

We disagree. As we state in response to major points 2-5, it would be unfair to previous researchers if we would not acknowledge their work on bacteria. We also note that reviewer #1 and other focus-group readers of our manuscript have found the introduction useful to give background about lichen biology and the history of their exploration that inform how the reader interprets the results — especially true for readers of a broad-scope journal who do not work with this system every day.

The major section of results deals with the functional profile of the detected bacteria (line 311 Functional profiles of the most commonly detected bacteria, line 342-367 Carbon usage, 368-377 Aerobic an oxygenic phototrophy, 378-388 Nitrogen fixation, 389-410 Cofactor and iron scavenging, 411-426 Carbohydrate degradation). But unless there is a proof that bacteria are symbiotic to lichens we are reading about which pathways do bacteria detected on surface of lichens have.

Isn’t this a circular argument? The implication is that we should not explore the pathways possessed by these bacteria unless we know they are symbionts, otherwise we are just exploring pathways in bacteria on lichens. But if we don’t explore their pathways, how would we ever know if syntrophies exist? Again, we take a microbiologist’s approach here and are taking stock of the usable information content of the metagenomes, which does not end with MAG taxonomy; we are also focused on claims already made in the published literature.

Discussion

Major part of discussion (lines 446-525) is focussed on proving why bacteria and algae may go undetected in lichens to support that bacteria are common occurrence (=symbiotic according to authors). They refer to studies which have and have not found Cystobasidiomycetes, photobionts etc. in lichens before. Regarding this, first this is of methodological relevance and does not require a major section in discussion and second still it only shows that bacteria are present.

We disagree — the inability of metagenomes to recover photobiont and yeast MAGs is definitely worth discussing as the use of metagenomes is increasing, often without full awareness of their limitations. We also feel that it is justifiable to analyze the high frequency bacterial content of metagenomes against the background of past claims.
